# *Caenorhabditis elegans* ETR-1/CELF has broad effects on the muscle cell transcriptome, including genes that regulate translation and neuroblast migration

**DOI:** 10.1186/s12864-021-08217-6

**Published:** 2022-01-06

**Authors:** Matthew E. Ochs, Rebecca M. McWhirter, Robert L. Unckless, David M. Miller, Erik A. Lundquist

**Affiliations:** 1 Program in Molecular, Cellular, and Developmental Biology, Department of Molecular Biosciences, University of Kansas, Lawrence, KS 66045 USA; 2 Department of Cell and Developmental Biology and Department of Biological Sciences, Vanderbilt University, Nashville, TN 37203 USA

## Abstract

Migration of neuroblasts and neurons from their birthplace is central to the formation of neural circuits and networks. ETR-1 is the *Caenorhabditis elegans* homolog of the CELF1 (CUGBP, ELAV-like family 1) RNA-processing factor involved in neuromuscular disorders. *etr-1* regulates body wall muscle differentiation. Our previous work showed that *etr-1* in muscle has a non-autonomous role in neuronal migration, suggesting that ETR-1 is involved in the production of a signal emanating from body wall muscle that controls neuroblast migration and that interacts with Wnt signaling. *etr-1* is extensively alternatively-spliced, and we identified the viable *etr-1(lq61)* mutant, caused by a stop codon in alternatively-spliced exon 8 and only affecting *etr-1* isoforms containing exon 8. We took advantage of viable *etr-1(lq61)* to identify potential RNA targets of ETR-1 in body wall muscle using a combination of fluorescence activated cell sorting (FACS) of body wall muscles from wild-type and *etr-1(lq61)* and subsequent RNA-seq. This analysis revealed genes whose splicing and transcript levels were controlled by ETR-1 exon 8 isoforms, and represented a broad spectrum of genes involved in muscle differentiation, myofilament lattice structure, and physiology. Genes with transcripts underrepresented in *etr-1(lq61)* included those involved in ribosome function and translation, similar to potential *CELF1* targets identified in chick cardiomyocytes. This suggests that at least some targets of ETR-1 might be conserved in vertebrates, and that ETR-1 might generally stimulate translation in muscles. As proof-of-principle, a functional analysis of a subset of ETR-1 targets revealed genes involved in AQR and PQR neuronal migration. One such gene, *lev-11/tropomyosin*, requires ETR-1 for alternative splicing, and another, *unc-52/perlecan*, requires ETR-1 for the production of long isoforms containing 3′ exons. In sum, these studies identified gene targets of ETR-1/CELF1 in muscles, which included genes involved in muscle development and physiology, and genes with novel roles in neuronal migration.

**Supplementary Information:**

The online version contains supplementary material available at 10.1186/s12864-021-08217-6.

## Introduction

Migration of neuroblasts and neurons is a key developmental process in the formation of neural circuits and networks. The CELF (CUGBP, ELAV-like family) class of RNA-binding proteins is implicated in a wide variety of neuromuscular and neurodegenerative disorders, including Myotonic Dystrophy type I (DMI) [1–7], the cardiac syndrome arrythmogenic right ventricular dysplasia [8], Alzheimer’s disease [9], spinocerebellar ataxia type 8, and possibly fragile X syndrome [10, 11]. CELF proteins control mRNA processing, including alternative splicing [12–14], and regulation of translation [15], and mRNA transcript stability [16, 17]. CELF protein structure is characterized by three RNA-recognition motifs (RRM) with a non-conserved region between RRM2 and RRM3, an RRM organization conserved across CELF protein family members [16].

Vertebrate genomes encode up to six CELF molecules [18], CELF1–6. In *C. elegans,* there are two *CELF* genes, *etr-1* (most similar to *CELF1–2*) [19, 20], and *unc-75* (most similar to *CELF3–6)* [21]. *unc-75* controls alternative splicing events predominantly in the nervous system [21–25]. ETR-1 is involved in muscle development, as knockdown of ETR-1 results in severe muscle disorganization and embryonic lethality [19]. ETR-1 also controls cell corpse engulfment in the germline [26], and influences neuronal migration non-autonomously from body wall muscle [20].

ETR-1 acts in muscle to guide the long-range migration of the Q neuroblast descendants in *C. elegans* [20]. The Q neuroblasts, QR and QL, are bilaterally symmetrical cells that undergo similar divisions and stereotypical migrations (reviewed in [27]). QR is born on the right side of the animal and QR descendants migrate anteriorly. QL is born on the left side of the animal and QL descendants migrate posteriorly. Both QR and QL produce three functional neurons, and two apoptotic bodies. QR produces AQR, AVM and SDQR, with AQR migrating the furthest, residing just posterior to the posterior pharyngeal bulb in the anterior deirid ganglion. QL produces PQR, PVM and SDQL, with PQR migrating the furthest, residing posterior to the anus in the phasmid ganglion [28–30]. Due to the stereotypical migrations, the Q neuroblasts are a powerful system to identify migration defects and study the genetic mechanisms controlling migration. Initial QL and QR migrations are controlled by interactions between three receptor molecules UNC-40/DCC, PTP-3/LAR and MIG-21, and the Fat-like Cadherins CDH-3 and CDH-4 [31–35]. Migration of the Q descendants is controlled by Wnt signaling along the anterior-posterior body axis [36–38]. Previous studies have implicated body wall muscle cells as sources of migration factors for the Q neuroblast descendants [39, 40].

The *etr-1(lq61)* mutation was isolated in a forward genetic screen for AQR and PQR migration defects [20]. *lq61* introduces a premature stop codon in alternatively-spliced exon 8, which is present in a subset of *etr-1* isoforms. Total knockdown of *etr-1* resulted in embryonic lethality with muscle defects [19], yet *etr-1(lq61)* animals are viable and fertile, consistent with *lq61* being a hypomorphic mutation. *Etr-1* is expressed in all cells of the embryo [41], but acts in the muscle cells in a non-autonomous manner to control AQR and PQR migrations [20]. Furthermore, *etr-1(lq61)* interacts genetically with *Wnt* mutations in AQR and PQR migration.

As *etr-1(lq61)* is a viable and fertile mutation, it presented a unique opportunity to identify gene targets of a CELF1/2 family member in muscles, and to define target genes that contribute to the non-autonomous control of AQR and PQR migration by ETR-1. We used fluorescence activated cell sorting of *C. elegans* body wall muscle cells from wild-type and *etr-1(lq61)* mutants, combined with RNA-seq, to define muscle-expressed genes with alternative exon usage and transcript accumulation and in *etr-1(lq61)* mutants. This analysis revealed genes involved in myofilament lattice assembly and adhesion, and muscle physiology. Genes with underrepresented transcripts in *etr-1(lq61)* were involved in translation and ribosome function. As proof of principle, a pilot functional screen identified new genes for AQR and PQR migration, including *unc-52/perlecan* and *lev-11/tropomyosin*. ETR-1 targets, including *lev-11/tropomyosin* and genes involved in translational and ribosome function, were also identified in vertebrates [16, 17], suggesting a deep evolutionary conservation of CELF targets and potentially conserved molecular mechanisms of CELF1/2 function from *C. elegans* to vertebrates.

## Results

### Fluorescent-activated cell sorting of muscle cells and RNA seq

**Fig. 1:**
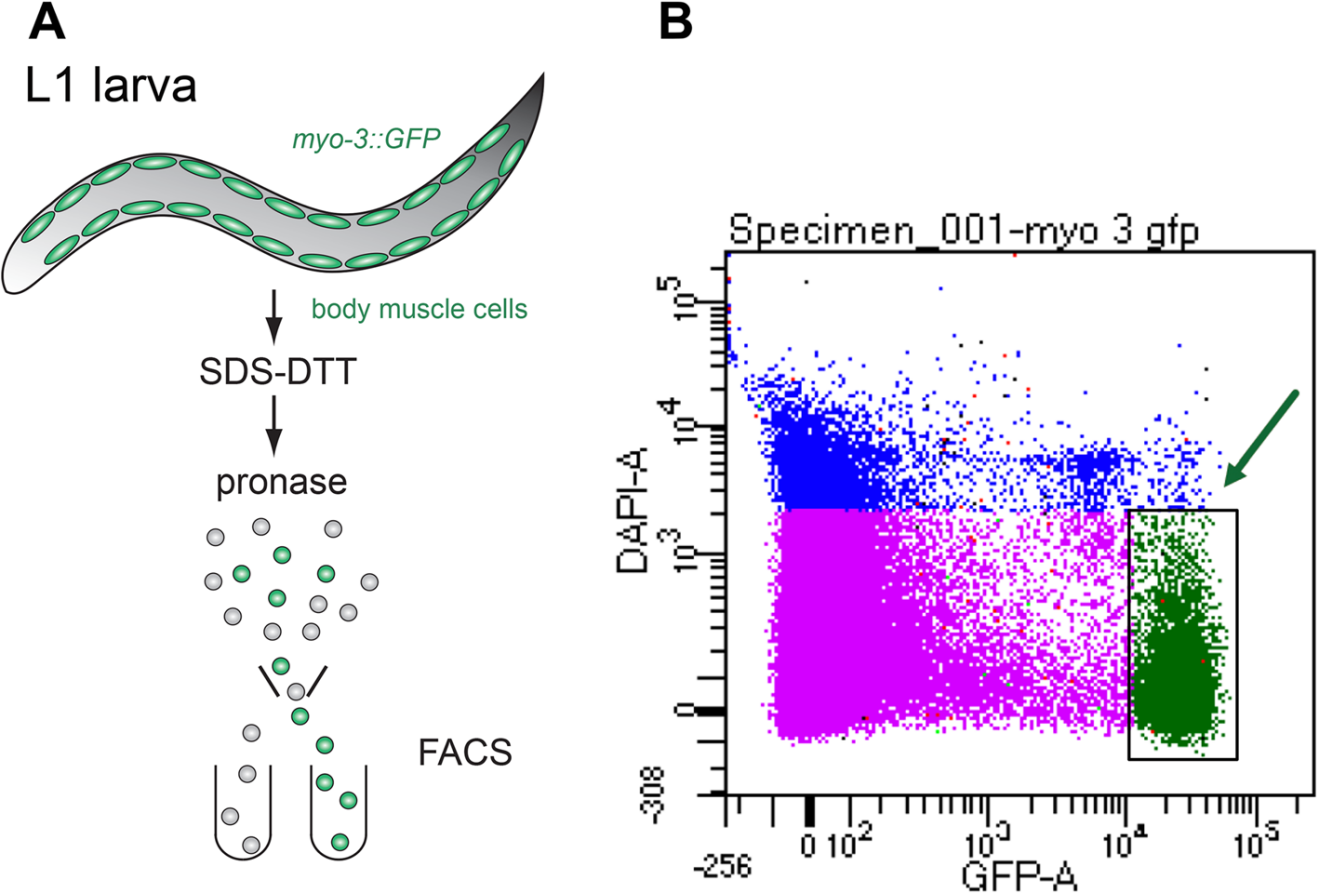
FACS isolation of body muscle cells from wild type and *etr-1(lq61)* mutant L1 larvae. **A** Synchronized L1-stage larvae were dissociated by successive treatments with SDS-DTT and pronase to release *myo-3::gfp* labeled body muscle cells for isolation by FACS. **B** FACS scatter plot. Viable and brightly labeled myo-3::GFP marked cells (arrow) were captured for RNA extraction. Damaged cells were excluded by DAPI staining

*myo-3::gfp*-expressing body wall muscle cells from synchronized early L1 larvae were isolated by FACS as described in Material and Methods and in [42, 43] (Fig. 1). Muscles were isolated from the wild-type (N2) strain and *etr-1(lq61)* mutants. Three biological replicates for each genotype were isolated. RNA was also isolated from triplicate samples of non-dissociated L1 larvae for the all-cell control group.

A total of 12 RNA-seq libraries were constructed, representing three biological replicates of both N2 and *etr-1(lq61)* with both sorted muscle cells and whole L1 larval stage cells (see Materials and Methods). Paired-end 150-bp reads were generated from each of the 12 samples using the Illumina Nextseq550 platform. FASTQ files can be accessed in the Sequence Read Archive, Project number PRJNA733501.

### A muscle cell transcriptome defined by RNA-seq

**Fig. 2:**
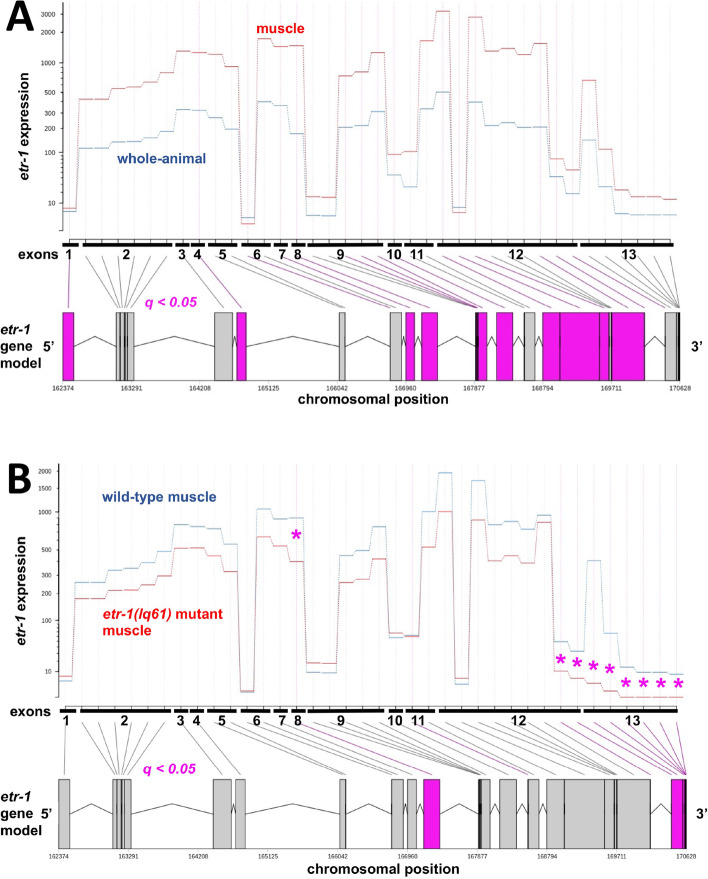
Output of DEXSeq showing differential exon usage. **A** Exon usage of *etr-1* comparing wild-type muscle to wild-type whole animal. The red line represents muscle expression, the blue line represents whole animal expression. Exons that are significantly different (*q* ≤ 0.05) are in purple and indicated with purple asterisks. *Etr-1* exons were overrepresented in muscle cells, including exon 8 harboring the *lq61* mutation. **B** Exon usage of *etr-1* comparing *etr-1(lq61)* muscle cells to wild-type muscle cells. The red line represents *etr-1(lq61)* expression and the blue line represents wild-type expression. *Etr-1* exons generally were underrepresented in *etr-1(lq61)*, with exon 8 and the 3′ exon significantly so (purple). Some exons have multiple comparisons because of different 5′ and 3′ exon boundaries for the exon in transcript isoforms annotated in the .gtf file. For example, exon 2 has six distinct 5′ and 3′ boundaries in different annotated isoforms, whereas exons 3 and 4 have a single 5′ and 3′ boundary in all annotated isoforms

We used DEseq2 to identify genes with significant differential expression in wild-type muscle cells compared to all L1 stage wild-type cells (Supplemental File 1). There were 3718 protein-coding genes with significantly higher expression in muscle cells compared to all cells (log2-fold change ≥0.5849 (1.5x); *q* ≤ 0.05), including many canonical muscle structure and function genes previously shown to be expressed in muscle (e.g. *unc-15/paramyosin, unc-54/myosin, unc-95/paxillin,* and the myofilament structure *pat* genes [44]. DEseq2 also identified *etr-1* as being more highly expressed in muscle (Supplemental File 1; log_2_fold change 2.21; *q = 9.93e*^*− 37*^). Differential exon usage using DEXseq showed that multiple *etr-1* exons showed significantly increased expression in muscle compared to all cells as a whole (Fig. 2 A). The *etr-1* locus is extensively alternatively spliced [20, 26], and isoforms with exon 8 are required in muscles for Q neuroblast descendant migration [20]. Exon 8 expression was increased in muscles compared to all cells as a whole (Fig. 2A), suggesting that isoforms with exon 8 might be more abundant in muscles compared to all cells as a whole. Expression of 8763 protein coding genes was significantly reduced in muscle cells compared to all cells (Supplemental File 1). Differential expression of non-coding RNAs and pseudogenes is presented in Supplemental File 1.

### Genes with exon usage affected by *etr-1(lq61)* encode molecules involved in myofilament lattice structure and attachment, and muscle physiology

CELF family proteins are known to regulate splicing [12, 45], and we endeavored to determine the effects of ETR-1/CELF on the muscle transcriptome, including splicing. We compared exon representation across the genome in wild-type and *etr-1(lq61)* mutant muscle cells using the Bioconductor package DEXseq [46] (see Materials and Methods). Across the genome, there were 242 protein-coding genes and seven non-coding RNA genes with at least one exon significantly differentially represented in *etr-1(lq61)* muscle compared to wild-type (*q* ≤ 0.05) (Supplemental File 2).

The *etr-1(lq61)* mutation is a premature stop codon in alternatively-spliced exon 8 [20]. Exon 8 was significantly underrepresented in *etr-1(lq61)* muscle compared to wild-type (Fig. 2B), suggesting that transcripts containing exon 8 are reduced in *etr-1(lq61)* muscle cells, as predicted. Exon 13 was also significantly underrepresented in *etr-1(lq61).* Possibly, transcripts with exon 8 might preferentially contain exon 13. Alternatively, ETR-1 containing exon 8 might be involved in the regulation of processing of *etr-1* exon 13.

**Fig. 3:**
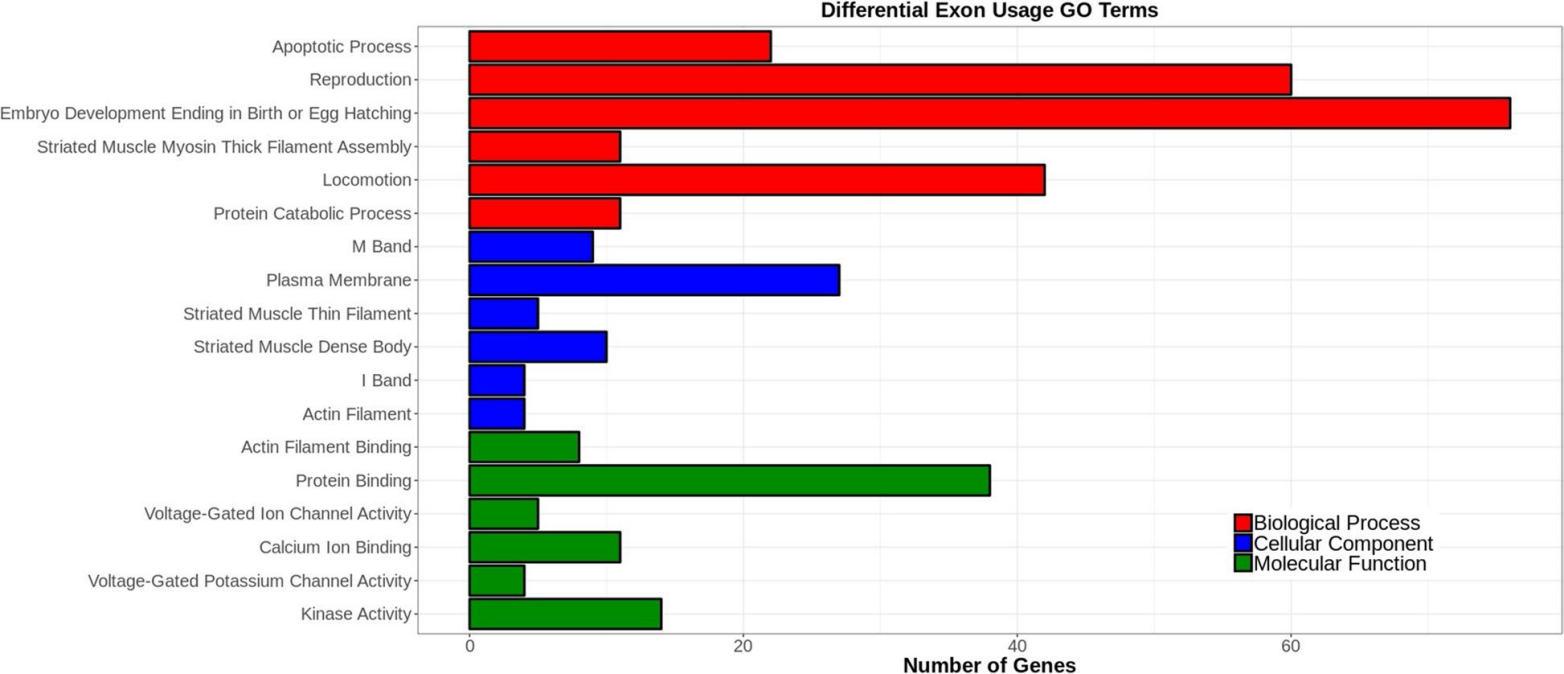
Gene ontology (GO) term analysis of genes with differential exon representation in *etr-1(lq61)*. Analysis was done using the Database for Annotation, Visualization and Integrated Discovery (DAVID) (see Materials and Methods). GO terms are along the Y axis, and the number of genes associated with each GO term is along the X axis. The six most significant GO terms (*q* ≤ 0.05) for three categories (biological process, cellular component, and molecular function) are shown. The entire GO term analysis is in Supplemental File 3

We used the Database for Annotation, Visualization and Integrated Discovery (DAVID) [47, 48] to perform a gene ontology term (GO term) analysis on this gene set that showed differential exon representation in *etr-1(lq61)* mutant muscle compared to wild-type muscle, including both over-and underrepresented exons in wild-type compared to *etr-1(lq61)* (see Materials and Methods). We analyzed GO terms for the three categories: biological process (BP), cellular component (CC) and molecular function (MF) (Supplemental File 3). The six most significantly enriched GO terms in each category are shown in Fig. 3. These include GO terms associated with myofilament lattice formation and function (e.g. striated muscle myosin thick filament assembly, locomotion, M band, striated muscle thin filament assembly, striated muscle dense body, I band, actin filament, and actin filament binding). Also included are muscle physiology GO terms (e.g. voltage gated ion channel activity, calcium ion binding, voltage gated potassium channel activity, and kinase activity). These are consistent with the previously-reported effects of *etr-1* RNAi knockdown on muscle development and attachment [19]. GO terms of apoptotic process and reproduction are also enriched, consistent with a known role of *etr-1* in germline development and engulfment of germ cell apoptotic corpses [26].

### Genes with transcript accumulation affected by *etr-1(lq61)* encode molecules involved in translation and ribosome function

The CELF-family proteins control transcript stability [12, 13, 45]. We used stringTie and DEseq2 to identify transcripts with differential accumulation in wild-type versus *etr-1(lq61)* muscle cells (see Materials and Methods). We identified transcripts that were differentially represented with a log_2_ fold change ≥1 (2x) and a false discovery rate less than 0.05 (*q* ≤ 0.05), to increase stringency given the large number of genes returned in this analysis (Supplemental File 4).

**Fig. 4:**
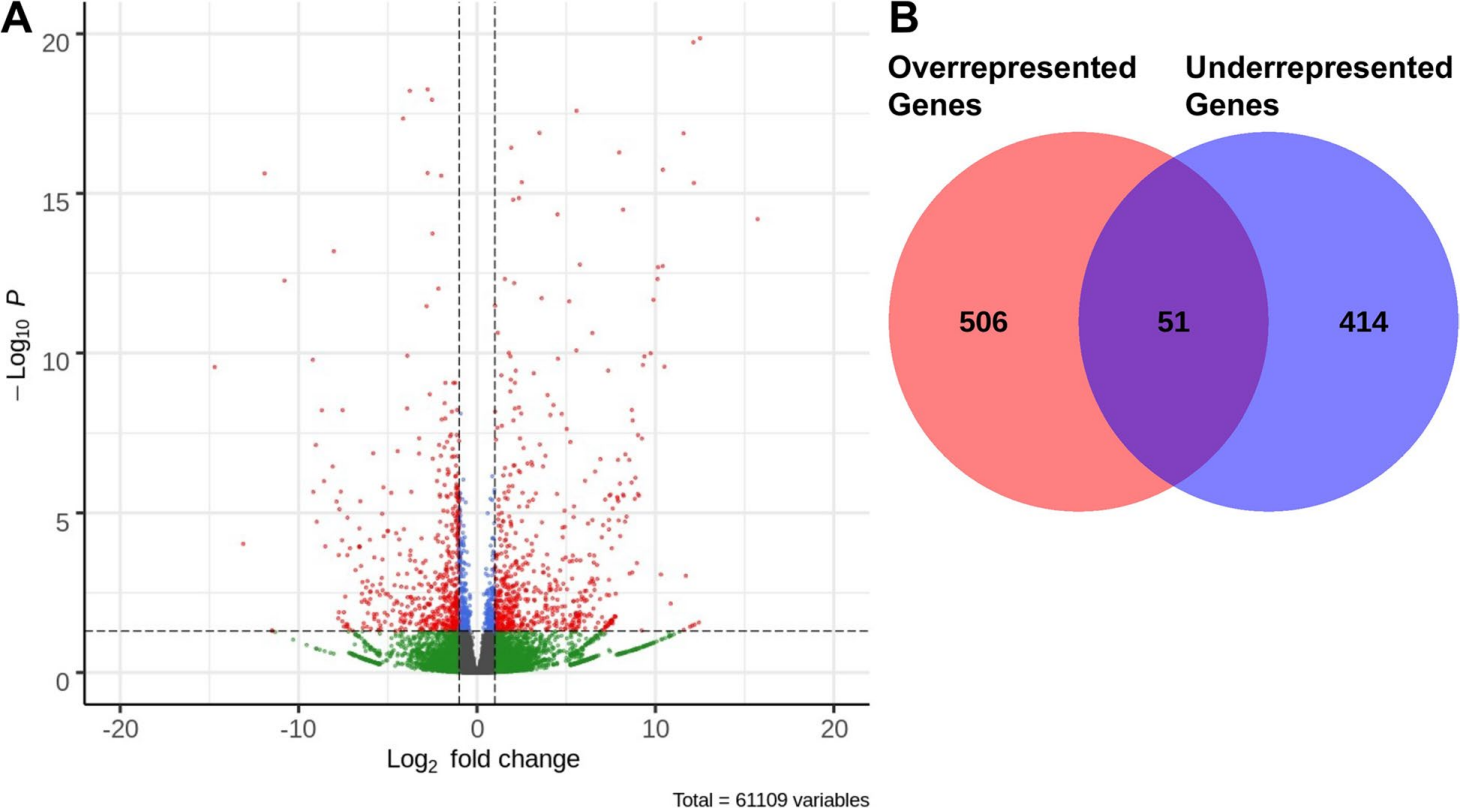
Differential transcript expression in *etr-1(lq61)* using DEseq2. **A** A volcano plot of differential transcript expression in *etr-1(lq61)*. Significant cutoff is *q* ≤ 0.05 and log_2_ fold cutoff is log_2_ fold > 1. Transcripts that meet these criteria are indicated by red dots. 630 transcripts were overrepresented, 550 transcripts were underrepresented. **B** Venn diagram of genes that had transcripts overrepresented or underrepresented (genes can have more than one transcript): 506 genes had transcripts that were only overrepresented in *etr-1* muscle cells; 414 genes had transcripts that were only underrepresented; and 51 genes had both over- and underrepresented transcripts

We identified 1180 transcripts representing 971 loci with altered accumulation in *etr-1(lq61)* (Fig. 4A), including coding and non-coding RNAs (Supplemental File 4). 506 loci had transcripts that were overrepresented, and 414 loci had transcripts that were underrepresented in *etr-1(lq61)* muscle cells (Fig. 4B). There were 51 loci in which some transcripts were overrepresented and some underrepresented (Fig. 4B and Table 1).

**Table 1:**
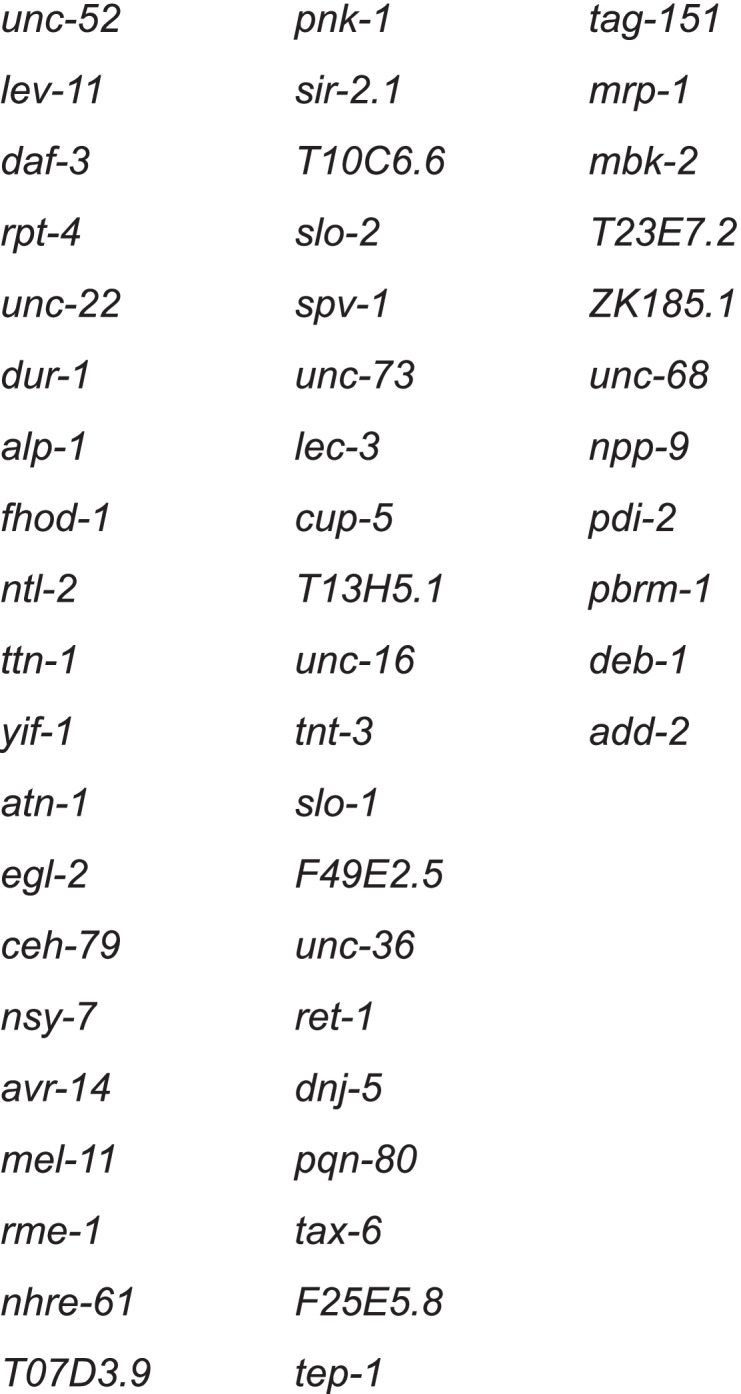
Genes with transcripts both overrepresented and underrepresented in etr-1(lq61)muscle

**Fig. 5:**
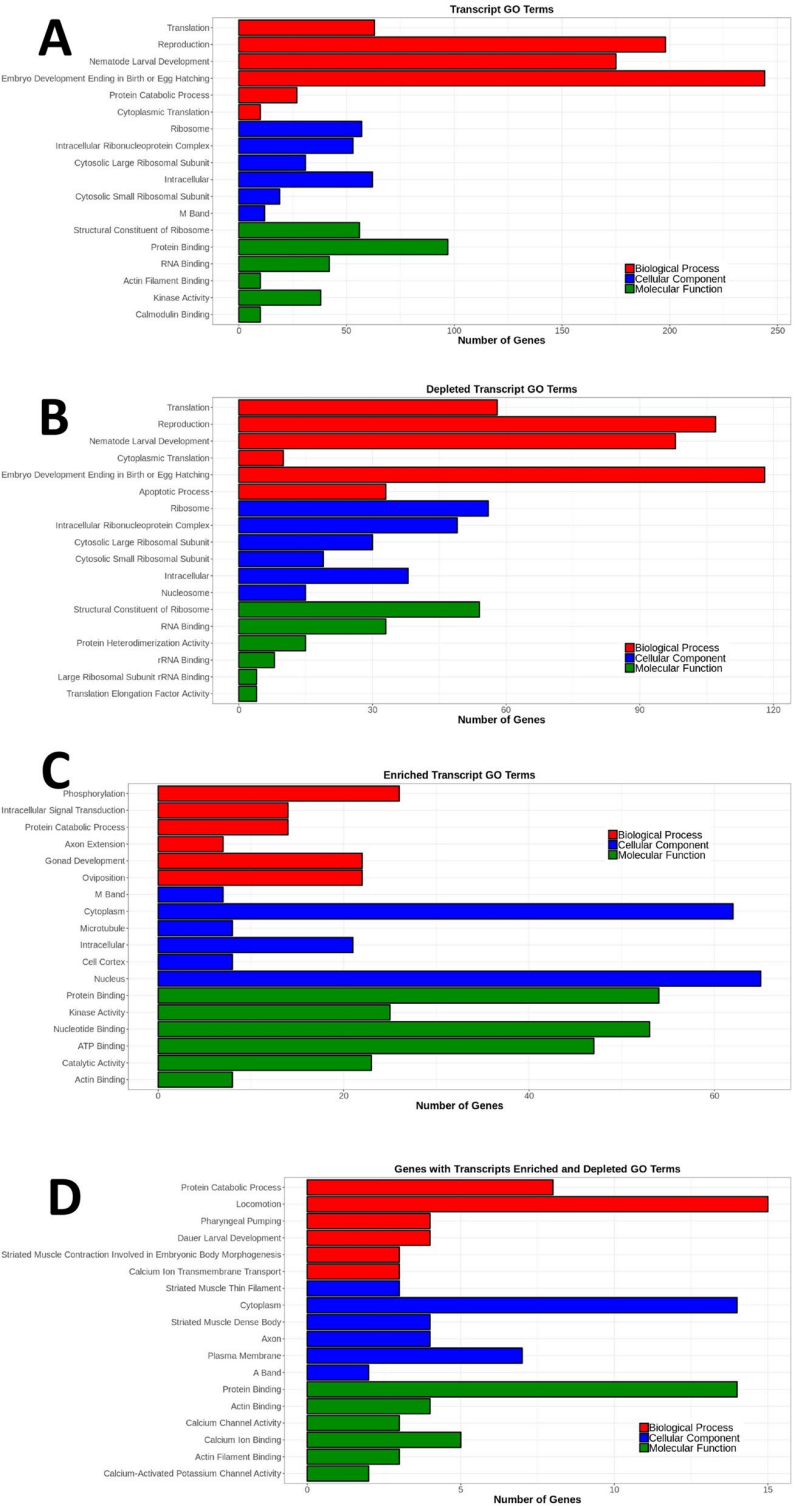
Gene ontology (GO) term analysis of genes with differential transcript representation in *etr-1(lq61)*. Figure is as described in Fig. 3. The six most significant GO terms (*q* ≤ 0.05) for three categories (biological process, cellular component, and molecular function) are shown. **A** All genes with transcripts affected by *etr-1(lq61).* **B** Genes with underrepresented transcripts. **C** Genes with overrepresented transcripts. **D** Genes with both underrepresented and overrepresented transcripts. The entire GO term analysis is in Supplemental File 5

Gene Ontology enrichment analysis was conducted on four separate groups of these muscle genes with transcripts affected by *etr-1(lq61)* (Supplemental File 5): all genes; genes with transcripts that were only underrepresented; genes transcripts that were only overrepresented; and genes that had transcripts both over- and underrepresented. The six most significant GO terms for each group are shown in Fig. 5. Considering all genes, GO terms associated with translation and ribosomal function were apparent, as well as myofilament structure, muscle physiology, reproduction, and embryonic and larval growth (Fig. 5A). Genes with transcripts underrepresented in *etr-1(lq61)* were described by GO terms representing translation and ribosomal function (10 of the 18 top GO terms) (Fig. 5B). Genes with overrepresented transcripts were described by GO terms representing a broad cross section of cellular function, but translation and the ribosome were not among these (Fig. 5C). Genes with both over- and underrepresented transcripts were described by GO terms representing myofilament lattice and muscle physiology, and other cellular functions (Fig. 5D). In sum, this GO term analysis suggests that *etr-1(lq61)* influences a broad spectrum of cellular events in muscle, including myofilament lattice and muscle physiology, as well as translation and ribosomal function. Notably, genes involved in translation and ribosomal function are strongly represented among those with transcripts reduced in *etr-1(lq61)*. A similar reduction of expression of genes involved in translation and ribosomal function was described after siRNA knock-down of *CELF1* in chicken cardiomyocytes [17].

### Genes identified by both differential exon usage and transcript accumulation

*etr-1(lq61)* affected transcript expression of 971 genes, and alternative exon representation of 244 genes. There were 102 genes shared between the 244 alternatively-spliced genes and the 971 genes that had differentially-expressed transcripts (Table 3), a significant association (*p* ≤ *0.0001)*. These might represent genes with transcripts for which ETR-1 controls both splicing and transcript accumulation. Alternately, the differential use of exons could influence the transcript accumulation DEseq2 algorithm, leading to under- or overrepresentation of transcripts by alternate exon usage. In any event, identification in both analyses suggests that ETR-1 might have strong effects on the transcripts of these genes.

### Genes affecting AQR and PQR migration

This analysis suggests that ETR-1 regulates multiple aspects of muscle cell function, most notably myofilament lattice, muscle physiology, and translation and ribosomal function. Previous studies indicated that *etr-1(lq61)* had a muscle-derived, non-autonomous effect on migration of AQR and PQR neurons [20]. Thus, ETR-1 might regulate a secreted signal from the muscles that directs AQR and PQR migration. Genetic analysis suggests this signal could act with or in parallel to Wnt signaling [20], which directs AQR and PQR migration [38].

We used feeding RNAi (see Materials and Methods) and mutants to knock down a subset of genes with known roles in cell migration, and genes that had transcripts both overrepresented and underrepresented in *etr-1(lq61)* muscles versus wild-type muscles (Table 1). AQR and PQR position in these animals was scored (see Materials and Methods) (Table 3). In *unc-52, unc-71,* and *skn-1,* between 2 and 5% of AQR and PQR neurons migrated in the wrong direction, as evidenced by AQR in position 5, and PQR in positions 1, 2, and 3. UNC-52 is the basement membrane heparan sulfate proteoglycan Perlecan [49] and will be discussed in more detail below. UNC-71 is an ADAM metalloprotease that has been shown to act in anterior Q descendant migration [50, 51]. SKN-1 is an ortholog of the human NFE2L1 transcriptional regulator that controls a wide variety of developmental events including muscle differentiation [52, 53]. Some genes displayed defects in the ability of AQR and PQR to migrate (> 10%), but not directional defects, as evidenced by AQR in positions 2, 3, and 4 and PQR in position 4. Many genes displayed few (< 10%) or no defects in the ability of AQR and PQR to migrate. Thus, some genes with transcripts that are regulated by ETR-1 in muscle had instructional roles in directing AQR and PQR migration, and some had permissive roles in the ability of AQR and PQR to migrate.

### The heparan sulfate proteoglycan UNC-52 affects AQR and PQR migration

*unc-52* encodes the basement membrane heparan sulfate proteoglycan Perlecan and is involved in myofilament lattice attachment to the basement membrane [49]. *unc-52* is extensively alternatively spliced [54, 55], including in the epidermis by CCAR-1 [56] and MEC-8 [57, 58] with consequences on hemidesmosome formation, muscle attachment and mechanosensory neuron function.

**Fig. 6.**
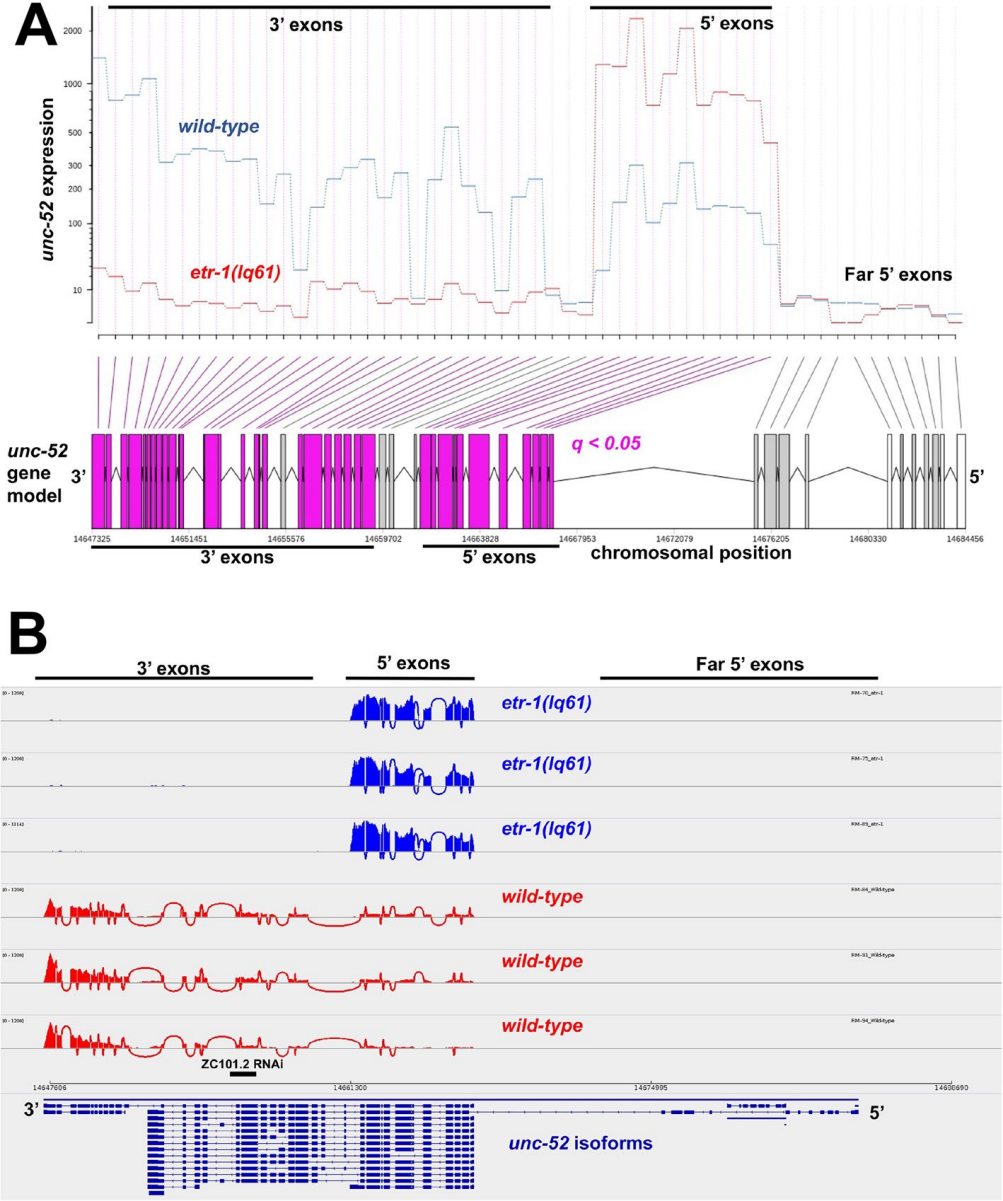
*etr-1(lq61)* affects *unc*-*52* exon usage. **A** DEXSeq output comparing *etr-1(lq61)* muscle to wild-type muscle as described in Fig. 2. In this plot, the 5′ end of the gene is to the right due to the gene being on the minus genomic strand in the .gtf file. **B** An IGV-Sashimi plot comparing *unc-52* exon usage in *etr-1(lq61)* muscle cells and wild-type muscles. The blue peaks represent *etr-1(lq61)* splice junctions in muscle cells in three independent biological replicates, and the red peaks represent splice junctions in wild-type muscles in three independent biological replicates

We found that *etr-1(lq61)* affected *unc-52* transcript expression in muscle cells. *unc-52* was identified in both exon representation by DEXseq and transcript accumulation by DEseq2 (Table 2). In *etr-1(lq61)* muscle cells, some 5′ exons were significantly overrepresented and 3′ exons significantly underrepresented compared to *wild-type* muscle cells. The far 5′ exons (see Fig. 6A) predicted for *unc-52* were not highly expressed in muscle in either background. We visualized *unc-52* splice junctions using the Sashimi plot function in the Integrated Genome Viewer (see Materials and Methods) (Fig. 6B). In wild-type animals, 3′ exons were well-represented (Fig. 6B). In *etr-1(lq61)*, 3′ exons were significantly underrepresented and 5′ exons significantly overrepresented. These data suggest that ETR-1 proteins that include exon 8 are required to produce the long isoforms of *unc-52* containing the 3′ exons in muscles.

**Table 2:**
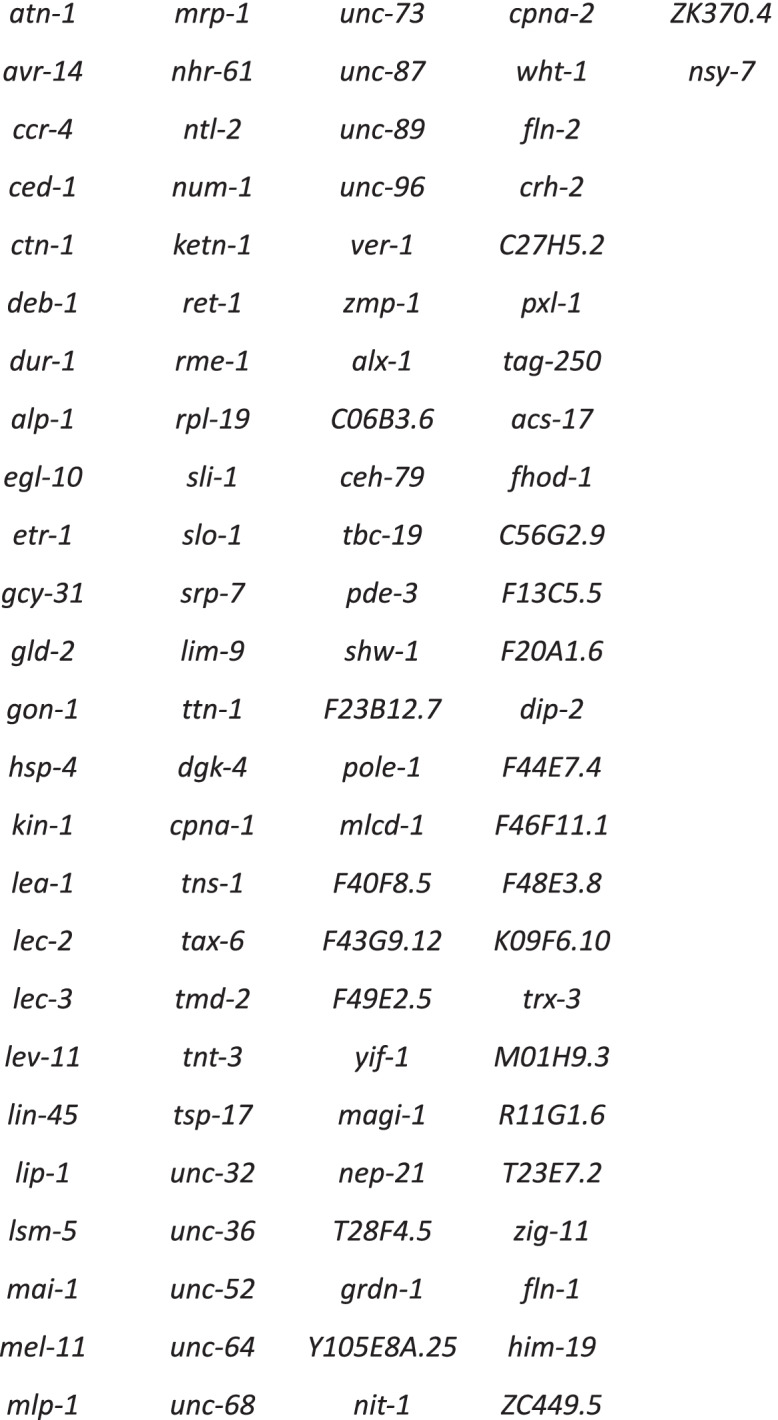
Genes with both exon representation and transcript accumulation affected by etr-1(lq61)

**Fig. 7:**
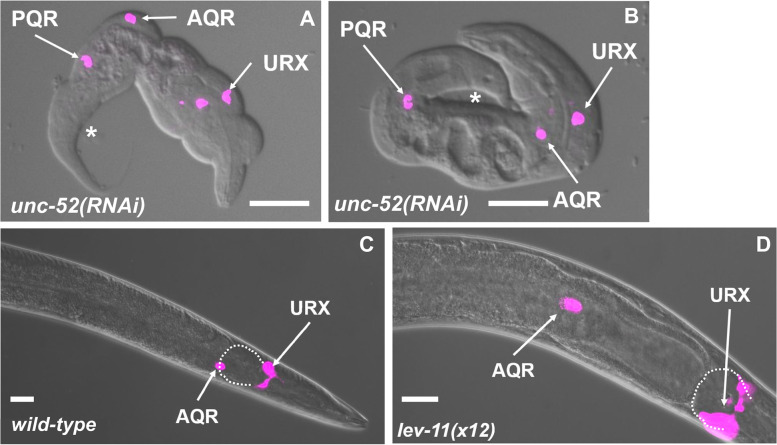
AQR and PQR defects in RNAi knockdown of *unc-52* and *lev-11*. Micrographs of merged DIC and fluorescence images are shown, with *gcy-32::cfp* expression in AQR and PQR (magenta). Two representative examples of paralyzed arrested at two-fold stage (Pat) *unc-52(RNAi)* animals are shown. The anus is indicated by an asterisk. **A** An *unc-52(RNAi)* animal displayed misplaced AQR and PQR. **B** An *unc-52(RNAi)* animal displayed a misplaced PQR, with AQR in the normal position. **C** The wild-type position of AQR just posterior to the posterior pharyngeal bulb (outlined with a dashed white line). **B** AQR position in a *lev-11(× 12)* mutant. AQR was displaced posteriorly approximately 50 μm from the posterior pharyngeal bulb. Scale bars represent 10 μm

As described above, *unc-52(RNAi)* resulted in AQR and PQR directional migration defects (Table 1). For RNAi, we utilized the ZC101.2 Source Bioscience II-9A20 clone, which is located located in the 3′ region of *unc-52*, the region that is underrepresented in *etr-1(lq61)* muscles (Fig. 6B). *unc-52(RNAi)* animals displayed the paralyzed, arrested at two-fold stage (Pat) phenotype similar to strong loss-of-function alleles of *unc-52* (Fig. 7 A and B) [59]. Notably, *etr-1(lq61)* animals, which do not express the 3′ exons from this *unc-52* region in muscle, do not show the Pat phenotype. The viability of *etr-1(lq61)* mutants could be.

due to a muscle-specific effect of ETR-1 on *unc-52*, with *unc-52* long isoforms with 3′ exons expressed and functional in other tissues (e.g. hypodermis) in *etr-1(lq61)* mutants. Alternatively, it is possible that the phenomenon of RNAi amplification [60] results in all *unc-52* isoforms being affected by RNAi, in which case conclusions about the roles of the 3′ exons cannot be drawn.

Despite embryonic lethality and the Pat phenotype, AQR and PQR were visible in *unc-52(RNAi)* arrested Pat animals. AQR and PQR displayed defects in the ability of AQR and PQR to migrate (Fig. 7 A and B) as well as defects in direction of migration (Table 3). In sum, ETR-1 proteins that contain exon 8 are required for the accumulation of long isoform transcripts of *unc-52* containing 3′ exons in muscles. Targeting *unc-52* with RNAi resulted in AQR and PQR defects, suggesting a role in AQR and PQR migration.

**Table 3:**
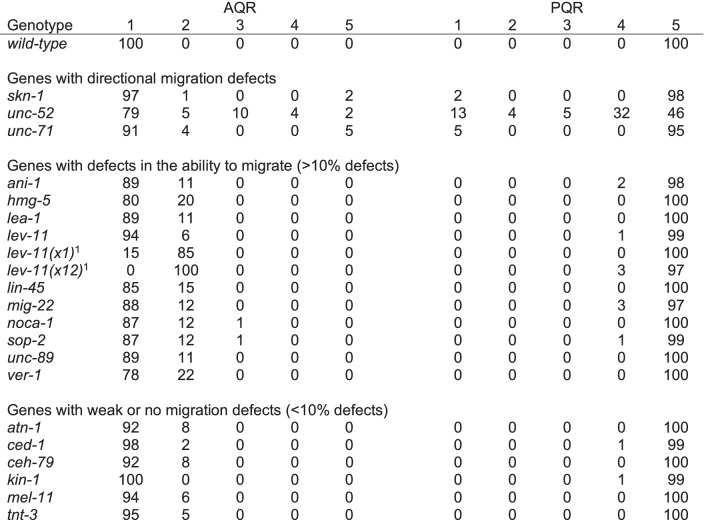
Candidate gene AQR and PQR migration defects using RNAi unless otherwise noted

^1^mutant alleles

### RNAi knockdown of *lev-11* results in AQR and PQR migration defects

*lev-11* was also identified by both exon representation and transcript accumulation in *etr-1(lq61)* (Table 2). *lev-11* encodes a tropomyosin [61], which is a known target of vertebrate CELF1 [16]. *lev-11* encodes multiple isoforms regulated in a tissue-specific manner [62, 63].

**Fig. 8.**
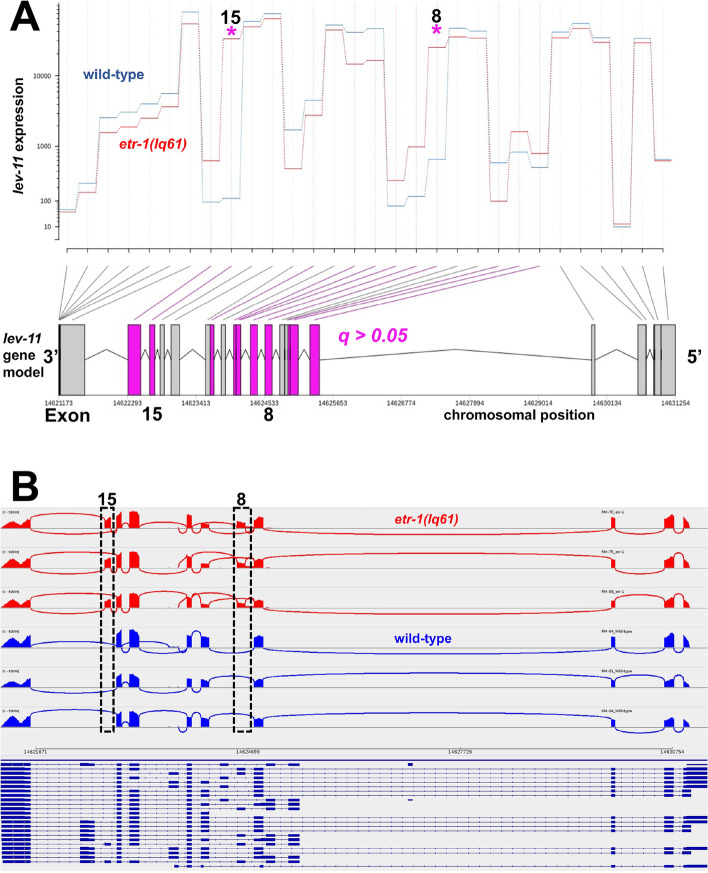
*etr-1(lq61)* affects *lev-11* exon usage. **A** DEXSeq output for *lev-11* comparing exon usage levels between *etr-1(lq61)* muscle and wild-type muscle, as described in Fig. 2. **B** IGV-Sashimi plot of *lev-11* splice junctions in wild-type and *etr-1(lq61*), as described in Fig. 6

*lev-11* exons 8 and 15 were significantly overrepresented in *etr-1(lq61)* muscle compared to wild-type muscle (Fig. 8A). IGV-Sashimi splice junction analysis revealed that *lev-11* exons 8 and 15 were included in *etr-1(lq61)* muscle and largely excluded in wild-type muscle (Fig. 8B). These results suggest that ETR-1 proteins with exon 8 controlled alternative splicing of *lev-11* in muscle cells, most notably removing exons 8 and 15 from *lev-11* transcripts.

*lev-11* RNAi resulted in AQR not migrating the full distance anteriorly, a phenotype also observed in *lev-11* mutants (Table 3 and Fig. 7 C and D). The region at which AQR stopped migrating in both *lev-11* RNAi and *lev-11* mutants was highly stereotyped, in position 4 just posterior to the normal position. These results suggest that *lev-11* might affect a specific developmental sign post rather than the general ability of AQR to migrate. In sum, our functional analysis indicates that genes with transcripts regulated by ETR-1 in muscles include those that control AQR and PQR neuron migration.

## Discussion

### Identification of ETR-1/CELF target genes via alternative exon usage and differential transcript abundance

CELF-family proteins regulate mRNA processing, including alternative splicing and transcript stability (reviewed in [16]). The *etr-1(lq61)* mutation presented a unique opportunity to determine CELF target genes in a specific tissue, body wall muscles of *C. elegans*. First, *lq61* corresponds to a premature stop codon in an alternatively spliced exon affecting only a subset of *etr-1* transcripts, and thus did not cause embryonic lethality [20]. Because *etr-1(lq61)* animals are viable, it was possible to isolate body wall muscle cells and their transcripts at a specific timepoint in L1 animals when Q neuroblasts are migrating. Second, *etr-1(lq61)* caused defects in AQR and PQR neuron migration in a cell-non-autonomous manner [20]. Thus, ETR-1 targets might be involved in producing a guidance signal for neurons from body wall muscle cells. Such an interaction might be conserved in vertebrate CELF function.

Two algorithms were used to compare differences in the transcriptomes of wild-type and *etr-1(lq61)* animals. DEXseq was used to assay alternative exon representation, and DEseq2 was used to assay differential transcript representation and splice junction usage. This analysis identified 244 genes with significant differential exon representation, and 1180 transcripts, corresponding to 971 genes, that were differentially represented. 102 genes were identified in both analyses, suggesting that these are not discrete categories and that exon abundance can affect predicted transcript abundance and vice versa. Our results suggest that a gene-by-gene approach using output from DEXseq, DEseq2, and IGV-Sashimi gives a clear picture of the effects on transcript structure and abundance.

It is likely that other ETR-1 targets were not identified in this analysis, as only a subset of *etr-1* isoforms containing exon 8 are affected by *etr-1(lq61)*. Exon 8 encodes a polyglutamine-rich region [20], which is thought to mediate interaction of CELF proteins with other splicing/RNA processing factors thus potentially altering the efficacy and specificity of the splicing reaction [64]. The genes identified in this analysis could be direct targets of ETR-1, or indirect targets through the effects of ETR-1 on other RNA processing genes.

### ETR-1 target genes include those involved in myofilament lattice assembly and function, muscle cell physiology, and translation

GO term analysis of ETR-1 target genes identified broad effects on the muscle cell transcriptome. The most significant GO terms described genes involved in myofilament lattice structure, assembly, and attachment were identified, along with genes involved in muscle cell physiology. This is consistent with the Pat phenotype of *etr-1* RNAi [19] and with the known effects of CELF family members in muscle cell physiology. GO terms involving germ cell and reproduction were also defined, consistent with a known role of *etr-1* in germ cell corpse apoptosis [26]. These genes might have conserved functions in the germline and in muscle.

Strikingly, the most significantly enriched GO terms describing genes with underrepresented transcripts in *etr-1(lq61)* involved translation and ribosomal function. Translation and ribosome GO terms were largely absent from the most significant GO terms describing genes with overrepresented transcripts and alternative exon usage, suggesting translation and ribosome GO terms were specifically associated with genes that require ETR-1 for transcript accumulation. This also suggests a general impairment of translation in *etr-1(lq61)* muscles. siRNA knock-down of *CELF1* in chicken cardiomyocytes also led to decreased expression of coding sequences with GO terms associated with translation and ribosomal function [17]. These parallel findings point to a potentially ancient evolutionary origin of CELF-family targets. Indeed, known targets of CELF molecules include tropomyosin, actinin, and troponin [16, 65]. Our analysis identified *lev-11/tropomyosin, atn-1/actinin,* and *tnt-3/troponin* as ETR-1 targets with differential alternative splicing events. Depletion of *etr-1* results in increased apoptotic bodies in the gonad, dependent upon the CED-1 cell corpse scavenger receptor [41]*.* Our analysis in muscles revealed a significant increase in the *ced-1* transcript in *etr-1(lq61)* (log_2_ fold change 1.58, *q* = 0.002), suggesting that the interaction with CED-1 might be conserved in muscles.

Other known CELF1 targets were not identified in our analysis. For example, *MTMR1* and *MTMR3*, encoding myotubularin-related proteins, are alternatively spliced by CELF1 during mouse heart development [12, 13, 66]. However, we did not detect significant differences in exon or transcript abundance of the related *C. elegans* genes, *mtm-1* and *mtm-3*. Possibly, *mtm-1* and *mtm-3* are not regulated by ETR-1 isoforms that contain exon 8. Alternately, ETR-1 might not regulate *mtm-1* and *mtm-3* in body wall muscle in *C. elegans*, as these genes were identified as CELF1 targets in cardiomyocytes [12, 13].

### Targets shared between ETR-1 and UNC-75

The CELF family of proteins contains six members in mouse and humans [16]. In *C. elegans* there are two members, ETR-1, similar to CELF1–2, and UNC-75, similar to CELF3–6. *unc-75* function is predominantly restricted to the nervous system [21–25]. Targets of UNC-75 in the nervous system include *unc-32*, which encodes for the α subunit of the V_0_ complex of vacuolar-type H^+^-ATPases [24], and *unc-64/syntaxin* [22, 23]. Both *unc-32* and *unc-64* were identified as ETR-1 targets in body wall muscle cells in our analysis. This finding suggests that ETR-1 and UNC-75 might regulate common targets in a tissue-specific manner (i.e. ETR-1 in muscle, and UNC-75 in neurons).

### ETR-1 targets control AQR and PQR neuronal migration

Previous studies showed that ETR-1 acts cell-non-autonomously in muscle to control AQR and PQR neuronal migration [20]. ETR-1 might be involved in the generation of a signal from body wall muscles that controls AQR and PQR migration. Body wall muscle is a known source of cues that control Q neuroblast lineage migration. SPON-1/F-spondin from posterior body wall muscles is required for robust AQR and PQR migration [40]. Furthermore, the NFM-1/NF-2 molecule acts non-autonomously, possibly in muscles, to control Q neuroblast protrusion and migration [39].

ETR-1 targets analyzed with the strongest effects on AQR and PQR migration include *skn-1, unc-71, unc-52*, and *lev-11*. The ADAM metalloprotease UNC-71 was previously implicated in anterior QR migrations [50, 51], and our results suggest that UNC-71 also controls directional PQR migration. SKN-1, UNC-52, and LEV-11 have not been previously implicated in Q lineage migration. The NFE2L1 transcriptional regulator SKN-1 is a known regulator of muscle differentiation [52, 53]. While RNAi knockdown of these genes in all tissues by feeding RNAi caused AQR and PQR migration defects, further studies will be required to show that ETR-1 regulation of these genes in muscle cells is relevant to AQR and PQR migration (i.e. these genes might act in other cells besides muscles to control AQR and PQR migration).

### *unc-52*/Perlecan is a target of *etr-1* and is required for AQR and PQR migration

*unc-52* encodes the basement membrane heparan sulfate proteoglycan Perlecan and is expressed in many different cell types, including the muscle cells [49, 58, 67]. *unc-52* has been shown to control the migrations of the distal tip cells [68]. The heparan sulfate epimerase HSE-5 has been shown to control Q neuroblast migration [69, 70], but loss of no single HSPG or in double mutant combination has been shown to affect Q migrations, including hypomorphic *unc-52* alleles [70].

*unc-52* was identified as a target of ETR-1 in muscles, with the 3′ exons of *unc-52* underrepresented in *etr-1(lq61)*. This finding suggests that ETR-1 with exon 8 is required for the accumulation of the long *unc-52* isoforms with the 3′ exons. Depletion of *unc-52* by RNAi resulted in embryonic lethality and severe AQR and PQR migration defects, including directional defects similar to *hse-5*. This finding suggests that UNC-52 might be the HSPG through which HSE-5 is controlling Q migration. Both *unc-52* and *hse-5* mutants display defects in AQR and PQR direction of migration, suggesting that UNC-52 is not merely a substrate for cell migration but rather provides directional information.

Our analysis showed that *unc-52* isoforms containing the 3′ exons were strongly underrepresented in *etr-1(lq61)* muscle, with the shorter ZC101.2b.1 transcript the predominant *unc-52* mRNA. The *unc-52* RNAi clone we used is located in these 3′ exons, suggesting that the long isoforms with the 3′ exons are important for AQR and PQR migration. The 3′ exons mainly encode for Immunoglobulin family domains, some of which are alternatively spliced in the epidermis by other factors, MEC-8 and CCAR-1 [56–58]. RNAi of *unc-52* resulted in embryonic lethality, yet *etr-1(lq61)*, which nearly eliminates transcripts with the 3′ exons in muscles, is viable. This is likely due to *unc-52* function in other tissues not regulated by *etr-1* (i.e. epidermis). Indeed, *mec-8* function in epidermis is sufficient to rescue *mec-8; unc-*52 synthetic lethality and the Pat phenotype [58]. This is consistent with the idea that *unc-52* long isoforms in muscle are not required for embryonic viability but are required for AQR and PQR migration.

### *lev-11* is alternatively spliced by *etr-1* and is required for AQR and PQR migration

We showed that ETR-1 exon 8 isoforms are required for alternative splicing of *lev-11* transcripts in muscle by the removal of exons 8 and 15 (Fig. 8B)*.* It was shown previously that CELF proteins regulate the alternative splicing of tropomyosin in chicken muscle development [71]. Isoforms of *lev-11* are expressed in a tissue-specific manner [62]. *lev-11* isoforms without exons 8 and 15 are expressed robustly in wild-type body wall muscle cells [62] (Fig. 8B), whereas those containing *lev-11* exons 8 and 15 are expressed in pharyngeal muscle and the excretory cell [62]. Our results suggest that ETR-1 with exon 8 is required to exclude exons 8 and 15 from *lev-11* transcripts in body wall muscle cells. Possibly, *lev-11* isoforms lacking exons 8 and 15 are optimized for wall body muscle structure or function.

Knockdown of *lev-11* by either RNAi or in *lev-11* mutants resulted in AQR migration defects, with most AQR cells stopping approximately 50 μm posterior to its normal position near the posterior pharyngeal bulb. This characteristic stopping point suggests that *lev-11* affects a developmental guidepost rather than generally affecting the ability of AQR to migrate, which results in stoppages of AQR along anterior body in positions 2, 3, and 4 (e.g. *epi-1/laminin* mutants) [72]. Two-hybrid studies suggest that LEV-11 physically interacts with MIG-14/Wntless [73], which is involved involved in Wnt secretion [74–76]. Mutation of *mig-14* results in Q neuroblast migration defects [37, 77, 78], including AQR and PQR directional defects [38]. *etr-1* interacts genetically with *Wnt* mutations to control AQR and PQR migration, either directly or through a parallel pathway [20]. Expression of three *Wnt* genes is significantly increased in body wall muscle (Supplemental File 1): *egl-20* (log2-fold change = 2.96), *cwn-1* (log2-fold change = 2.74), and *cwn-2* (log2-fold change = 1.02), but their exon use and transcript levels are not affected by *etr-1(lq61)*. Intriguingly, *cwn-1* mutants display posteriorly-displaced AQR similar to but weaker than *lev-11* mutants [38]. It is plausible that *etr-1* regulates the splicing of *lev-11* in muscle to control an interaction with MIG-14. Disrupting this interaction might result in improper production of a Wnt signal, causing AQR and PQR migration defects. Further studies will be aimed at exploring this mechanism.

### Linking the effects of *etr-1(lq61)* on the muscle cell transcriptome to mutant phenotypes

Here we define transcripts in body wall muscle cells that are affected by mutation of the CELF1 family member *etr-1*. The *etr-1(lq61)* mutation selectively disrupts isoforms that include exon 8, leaving other ETR-1 isoforms intact. Thus, this set of body muscle transcripts include only those affected by ETR-1 with exon 8. *etr-1(lq61)* affected splicing and accumulation of transcripts of genes involved in a broad spectrum of muscle cell function including myofilament structure and attachment and physiology. Strikingly, genes involved in translation and ribosomal function were abundant among genes with transcripts underrepresented in *etr-1(lq61)*. siRNA knock-down of *CELF1* in chicken cardiomyocytes also led to a decrease in genes with GO terms associated with translation and ribosomal function [17], suggesting a deep evolutionary conservation of CELF1 target genes. Indeed, *lev-11/tropomyosin, atn-1/actinin,* and *tnt-3/troponin* were also conserved targets in *C. elegans* and vertebrates [16, 65].

How do these broad effects on the muscle transcriptome result in *etr-1(lq61)* mutant phenotypes, including AQR and PQR migration? We demonstrate that genes identified here include those that control AQR and PQR migration, such as *unc-52* and *lev-11*. What is less clear is how the *etr-1-*specific effects on these transcripts in muscle cells contributes to AQR and PQR migration defects. It seems unlikely that the AQR and PQR defects in *etr-1(lq61)* result from disruption of one or a small handful of genes. Rather, more likely is that subtle effects on multiple genes controlling AQR and PQR migration contribute to the phenotype, such as *unc-52, lev-11*, and others. Future studies will be aimed at addressing this question.

## Materials and methods

### Strains and genetics

*C. elegans* strains were was cultured using standard methods at 20 °C. Wild-type strain was N2 Bristol. Alleles used were: LGII: *etr-1(lq61)*, *lqIs244[Pgcy-32::cfp], lex-11(× 1 and × 12).* LG V: *lqIs58[Pgcy-32::cfp].* LG unknown: *ccIs4521[myo-3p::GFP::LacZ::NLS + (pSAK4) myo-3p::mitochondrial GFP + dpy-20(+)]* [79]*.*

### Larval disruption for cell sorting

For body wall muscle cell sorting, the *myo-3::gfp* transgene *ccIs4521* was used, in *wild-type* and *etr-1(lq61)* backgrounds. *C. elegans* strains were grown on twenty × 150 mm 8P nutrient agar plates (for 1 l: 20 g bactopeptone, 3 g NaCl, 25 g agar) with *E. coli* strain NA22, which produces a thick bacterial lawn. Early L1 animals were synchronized by first collecting embryos using bleach (hypochlorite) treatment of gravid adult hermaphrodites, and allowing the embryos to hatch overnight into sterile M9 medium at 20 °C. This produced approximately 3 million starved, synchronized early L1 larvae. The Q neuroblasts of starved L1s were variably arrested in their early development, from initial migration to the first division after migration (data not shown). Preparations of dissociated larval cells were generated as previously described using SDS-DTT and Pronase treatment coupled with mechanical disruption using a pipet [42, 43].

### FACS analysis

Fluorescence activated cell sorting of *myo-3::GFP-*expressing body wall muscle cells was performed as previously described using a BD FACSAria with a 70 μm diameter nozzle [42, 43]. DAPI was added to mark dead or damaged cells which were excluded from each sample of viable myo-3::GFP labeled muscle cells. Profiles of GFP strains were compared to a non-transgene-bearing N2 standard to exclude auto-fluorescent cells. Cells were sorted into Trizol LS. RNA was extracted from the aqueous phase using 5PRIME phase lock gel heavy tubes and purified on a spin column (Zymo, R1013). 30,000–50,000 FACS-sorted cells from each experiment, yielded 5–100 ng total RNA which was used in RNA seq library preparation. Three independent replicate samples were obtained for both wild-type and *etr-1(lq61)* mutant animals.

### mRNA library preparation and sequencing methods

For an all-cells control group, total RNA was isolated from an aliquot of ~ 25,000 L1 larvae set aside before cell dissociation and FACS for each one of the samples. L1 larvae were quickly frozen in liquid nitrogen in a mortar and pestle and ground to powder. Trizol LS was added to the powdered animals for RNA extraction. Muscle FACS and whole L1 RNA extraction resulted in twelve RNA samples representing three biological replicates of N2 and *etr-1(lq61)* sorted muscles and three biological replicates of N2 and *etr-1(lq61)* whole L1 larvae all-cell controls. Quality of total RNA samples was ensured using an Agilent TapeStation, and quantity was determined using Qubit fluorimetry. Sequencing libraries were constructed with 5-100 ng of total RNA using the NEBNext Ultra II Directional RNA Library Kit for Illumina. The sequencing library construction process aimed for 300-bp inserts and included mRNA purification with poly-A beads, fragmentation, strand specific cDNA synthesis, end repair, 3′ end adenylation, adapter ligation, and PCR amplification. The constructed sequencing libraries were validated and quantified with Qubit and TapeStation assays. Each library indexed and sequenced in multiplex on the Illumina NextSeq550 system, generating paired-end, 150-base sequence reads from the libraries. Between 33 million and 39 million reads were generated for each of the 12 samples. Base calling was carried out by the Nextseq550 instrument Real Time Analysis (RTA) software. The base call files (bcl files) were demultiplexed and converted to compressed FASTQ files by bcl2fastq2.

### Data availability statement

All data are represented in this manuscript and all strains and reagents are available upon request. FASTQ files for this project are available in the Sequence Read Archive (SRA) under project number PRJNA733501. Computational code can be found in Supplemental Materials.

### RNA-Seq analysis

The *C. elegans* reference genome was downloaded from the following URL: //hgdownload.cse.ucsc.edu/goldenPath/ce11/bigZips/chromFa.tar.gz. Quality control of reads was performed using FastQC (version 0.11.5) and fastp (options: --detect_adapter_for_pe –length_required 75 –trim_front1 10 –trim_front2 10 –cut_mean_quality 25 –cut_window_quality 5 -cut_tail, version 0.19.8) [80]. Splicesite and exon models were built using the *C. elegans* release 11 GTF file and HISAT2 with default settings (hisat2_extract_splice_sites.py and hisat2_extract_exons.py). Reads were aligned to the genome using HISAT2 with default settings (version 2.1.0) [81]. Samtools (version 1.7) [82] was used to convert SAM files to BAM files, and to sort the BAM files for downstream analysis. Read counts were assembled using stringTie (version 1.3.5) [83, 84], and FeatureCounts (version 1.6.0) [85]. We used the accompanying Python script from stringTie to prepare the output specifically for use in DESeq2 [86].

### Differential exon usage analysis

Alternative splicing differences were determined by quantifying differential exon usage using the Bioconductor package DEXSeq (version 1.30.0) [46] in R. Expression profiles were built using FeatureCounts [85]. Exons that showed differential expression with an adjusted *p* value false discovery rate less than 0.05 were considered differentially expressed in our analysis. Briefly, in DEXseq, exon or exon bin representation is normalized against the total number of aligned reads in the sample using the estimateSizeFactors function, and a χ ^2^ test with dispersion estimates and corrected for multiple testing is used to derive a *q* value significance.

### Differential transcript expression analysis

We examined all cells versus muscle cells, and wild-type versus *etr-1(lq61)* muscle cells in separate analyses. Differential transcript expression was tested using the Bioconductor package DESeq2 (version 1.24.0) in R. Expression profiles were prepared using stringTie [83, 84], and the accompanying Python script was used to prepare a table for DESeq2. For both analyses, transcripts with a false-discovery rate adjusted *p*-value less than 0.05 were considered significantly differentially expressed. A similar statistical framework is used in DEseq2 as is used in DEXseq described above (χ^2^ with dispersion estimates and correction for multiple testing). Preparation of tables and graphs was carried out in R [87].

### Gene ontology analysis

Analysis was done using the Database for Annotation, Visualization and Integrated Discovery (DAVID: https://david.ncifcrf.gov/) [47, 48]. Alternative exon usage and alternative transcript expression were analyzed for biological process, cellular component, and molecular function separately. Gene lists were analyzed for terms that were significantly enriched at *q > 0.05* against all *C. elegans* genes. The complete GO term list for each separate test are in Supplementary files 3 and 5. Graphs were prepared in R [87].

### Sashimi plots

Sashimi plots were created using the Integrative Genomics Viewer (IGV), version 2.5.0 [88, 89]. Indexes to visualize the splice junctions on IGV were built using the samtools (version 1.7.0) index function [82].

### RNA-mediated gene interference (RNAi)

RNAi was administered via feeding, following standard protocols and clones from the Source BioScience library (Nottingham UK) [90, 91]. (Kamath et al., 2003). For each RNAi experiment we grew wild-type animals expressing *Pgcy-32::cfp* to visualize AQR and PQR on RNAi bacteria [92]. For each independent set of RNAi experiments, *ceh-20(RNAi)* was used as a positive control, as RNAi of *ceh-20* results in robust AQR and PQR defects [92].

### Scoring AQR and PQR migration defects

AQR and PQR migration was scored using *gcy-32::cfp* as previously reported [20, 30, 31]. Briefly, L4 animals were collected, mounted onto a 2% agar pad and immobilized by 5 mM of NaN_3_. Five positions along the length of the animal were noted. Position 1 is the normal AQR position in the head just posterior to the posterior pharyngeal bulb; position 2 is posterior to the normal position but anterior to the vulva, position 3 is proximal to the vulva both anteriorly and posteriorly, position 4 is the normal birthplace of QR and QL, and position 5 is the normal location of PQR, just posterior to the anus. 100 animals were scored for each RNAi clone.

## Supplementary Information

**Additional file 1.**

**Additional file 2.**

**Additional file 3.**

**Additional file 4.**

**Additional file 5.**

**Additional file 6.**

**Additional file 7.**

**Additional file 8.**

**Additional file 9.**

## Data Availability

All data are represented in this manuscript and all strains and reagents are available upon request from the corresponding author (erikl@ku.edu). FASTQ files for this project are available in the Sequence Read Archive (SRA) under project number PRJNA733501 (https://www.ncbi.nlm.nih.gov/sra/?term=PRJNA733501). Computational code can be found in Supplemental Materials.
